# Intergenerational educational mobility is associated with cardiovascular disease risk behaviours in a cohort of young Australian adults: The Childhood Determinants of Adult Health (CDAH) Study

**DOI:** 10.1186/1471-2458-10-55

**Published:** 2010-02-02

**Authors:** Seana L Gall, Joan Abbott-Chapman, George C Patton, Terence Dwyer, Alison Venn

**Affiliations:** 1 Menzies Research Institute, University of Tasmania, Hobart, Tasmania, Australia; 2 Faculty of Education, University of Tasmania, Hobart, Tasmania, Australia; 3 Murdoch Childrens Research Institute, Melbourne, Australia

## Abstract

**Background:**

Although educational disparity has been linked to single risk behaviours, it has not previously been studied as a predictor of overall lifestyle. We examined if current education, parental education or educational mobility between generations was associated with healthy lifestyles in young Australian adults.

**Methods:**

In 2004-06, participant and parental education (high [bachelor degree or higher], intermediate [vocational training], low [secondary school only]) were assessed. Educational mobility was defined as: stable high (participant and parent in high group), stable intermediate (participant and parent in intermediate group), stable low (participant and parent in low group), downwardly (lower group than parent) and upwardly (higher group than parent) mobile. We derived a lifestyle score from 10 healthy behaviours (BMI, non-smoking, alcohol consumption, leisure time physical activity and six components of diet). Scores >4 indicated a high healthy lifestyle score. We estimated the likelihood of having a high healthy lifestyle score by education (participant and parent) and educational mobility.

**Results:**

Complete data were available for 1973 participants (53% female, age range 26 to 36 years). Those with lower education were less likely to have healthy lifestyles. Parental education was not associated with having a high healthy lifestyle score after adjustment for participant's education. Those who moved upward or downward were as likely to have a high healthy lifestyle score as those in the group they attained.

**Conclusions:**

We found clear disparities in health behaviour by participant education and intergenerational educational mobility. People attaining a higher level of education than their parents appeared protected from developing an unhealthy lifestyle suggesting that population-wide improvements in education may be important for health.

## Background

Some researchers have shown that socioeconomic inequalities in health can be accounted for by a higher prevalence of unhealthy behaviours in those of lower socioeconomic status (SES) [1], but findings are not consistent [2,3]. By extending our understanding of the association between SES and the behaviours that contribute to disease, we may find ways to reduce health inequalities. Increasing participation in education may be one approach, as demonstrated by improvements in maternal education leading to reduced infant mortality [4]. Intergenerational social mobility, i.e. a change in SES between parents and offspring, has been associated with mortality [5,6] and we recently demonstrated that upward social mobility was associated with increasing physical activity and fitness over a 20-year period [7]. However, the importance of such mobility for other aspects of health remains uncertain [8].

Previous research on socioeconomic inequalities in health behaviour has had several limitations. First, most investigators examined single risk behaviours as noted recently [9]. A focus on individual health behaviours remains important in designing behaviour-specific interventions that may reduce inequalities. However, a focus on how behaviours occur together will increase our understanding of the absolute burden in disease risk across socioeconomic strata. The recent development of a lifestyle score that comprises healthy behaviours has simplified this process [10]. This score is useful because, in addition to reporting on behaviours, it predicts mortality [10] and is associated with biomedical cardiovascular risk factors in young adults [11]. This score is particularly appealing because it comprises 10 healthy behaviours that individuals can measure themselves without invasive or costly testing. Furthermore, all items align with recommendations from peak bodies, such as the National Health and Medical Research Council in Australia, and are evidence-based. The score sums the healthy behaviours, giving a total score out of 10. This method of scoring acknowledges the co-occurrence of risk behaviours, while keeping the calculation simple and accessible to the general population.

The second limitation is that most research into socioeconomic inequalities in health behaviours has included middle or older aged individuals, with limited focus on younger people. Including younger individuals is important because it helps limit reverse causation whereby poor health contributes to lower SES. Some have argued that research into socioeconomic inequalities in health is context specific, suggesting that studies are needed across regions and time periods [12,13]. Data from contemporary cohorts and regions other than Europe are particularly lacking.

The current study focuses on a national cohort of young Australian adults who originally participated in a school-based health and fitness survey in 1985. The aims of our study were to examine the associations of current education level, parental education at baseline, and intergenerational educational mobility with a lifestyle score based on healthy behaviours at follow-up.

## Methods

### Participants

The Childhood Determinants of Adult Health (CDAH) study is a 20-year follow-up of participants in the 1985 Australian Schools Health and Fitness Survey (ASHFS) [14]. Of the 8498 7 to 15 year-olds who participated in the ASHFS, 5170 were enrolled in the CDAH study. For those that did not enrol, 20% (n = 1658) were unable to be traced, 10% (n = 817) did not respond to contact, 9% (n = 767) refused to participate, and 1% (n = 86) were deceased. Of the 5170 that enrolled, 2900 completed questionnaires on education and health behaviours and 2410 attended study health clinics, where we measured BMI. The number of participants with complete data is lower than that enrolled in the CDAH study largely due to the burden of attending a study clinic, which involved approximately three hours of testing, and the distance needed to travel to attend clinics. The current analysis includes participants (aged 26 to 36 years) who had both questionnaire and BMI data (n = 1973). The final number included in some analyses is less than this due to missing data for some covariates.

Those with complete follow-up data (n = 1973) were older (p < 0.001), more often female (p < 0.01), from higher SES postcodes [15] (p < 0.01) and had a lower mean BMI (p < 0.01) in 1985 than those without complete data (n = 6525, data not shown). Compared with the Australian population of a similar age from the National Health Survey [16] and Census [17], the 1973 people included were more likely to be married or living as married (70% versus 62%), have post-secondary school education (75% versus 52%), be low risk consumers of alcohol (93% versus 87%) and consume low fat milk (52% versus 36%) but had similar levels of never smoking (55% versus 51%), normal weight status (47% versus 49%) and recommended fruit (45% versus 47%) or vegetable consumption (10.5% versus 10.5%).

### Socioeconomic position

We used education level to indicate socioeconomic position [18]. Participants reported their own highest level of education and, retrospectively, their mother's and father's highest level of education when the participant was 12 years old. Parental education was classified according to the highest level achieved by either parent, a method used by other investigators [19,20]. Participant and parental education was collapsed into three categories: low, included all schooling up to completion of high school; intermediate, including trades and apprenticeships; and high, included bachelor degrees or higher. The Australian primary and secondary education system comprises both public and private institutions. In 2008, 63% of students attended public schools, which do not have tuition fees [21]. The funding of tertiary education has changed between the parental and participant generations in our study. In the early 1970s, the Australian government abolished tuition fees for tertiary education [22]. In 1989, the system changed and tuition fees were deferred using an interest free loan. The loan is then repaid through the tax system once a person reaches a certain income threshold.

### Educational mobility

We used parental and participant education to assign educational mobility into one of five categories [7]. These were: stable high (parent and participant had tertiary education), stable intermediate (parent and participant had vocational training), stable low (parent and participant had achieved high school education only), downward mobility (participant's education was lower than parent's education), and upward mobility (participant's education was higher than parent's education).

### Healthy lifestyle score

We used a lifestyle score that has been shown to predict mortality in elderly men [10]. The score originally developed by Spencer and colleagues comprised eight items; however, the score was recently revised to include fruit and vegetable consumption and the type of spreads used on savoury items, such as bread [23]. The score therefore comprises 10 items: body mass index (BMI) <25 kg/m^2^, never smoker or ex-smoker ≥ 12 months, ≥ 3 hours of moderate to vigorous leisure time physical activity (LTPA) per week, ≥ 20 grams of alcohol per day, fish consumption ≥ three times per week, consuming red meat < five times per week, consumption of ≥ two serves of fruit and ≥ five serves of vegetables per day (one item), regular use of skim milk, use of margarine instead of butter and not adding salt to food. BMI was calculated from height and weight measured by trained data collectors. LTPA was measured using the international physical activity questionnaire-long form, which has good reliability (r = 0.81 across 12 countries) and criterion validity (r = 0.33) [24]. Questionnaires gathered smoking, alcohol and dietary data. Consumption of at least two serves of fruit and five serves of vegetables per day was determined from two questions on usual daily consumption of these items. Weekly red meat consumption was derived from responses to 10 items and fish consumption from 9 items. People who responded 'never/rarely' to adding salt during and after cooking were classified as not adding salt to food. The use of margarine was determined from a question asking what type of spread the participant usually used on savoury items. Daily alcohol consumption in grams was estimated from the usual frequency of consumption (options ranged from 'never or less than once per month' to 'six times per day') of 10 alcohol beverages over the previous 12 months multiplied by the average alcohol concentration of each beverage [25].

The healthy lifestyle score was calculated by assigning a point for each behaviour. These were then summed, giving a total score ranging from 0 (no healthy behaviours) to 10 (all healthy behaviours).

### Covariates

We considered the following covariates: age, marital status (married/living as married and single/divorced/separated); history of cardiovascular disease or diabetes in participants (defined as self-report of any of myocardial infarction, stroke, angina or diabetes) or their family (defined as any of father or brother with myocardial infarction before age 50, or diabetes in any immediate family member) and the participant's area of residence (three categories: major city, inner regional and outer regional/remote location) [26].

### Statistical analysis

We examined the prevalence of individual items in the lifestyle score by participant and parental education as well as educational mobility. As other authors have done [10], we dichotomised the healthy lifestyle score at the median. This resulted in two groups: high (scores from 5 to 10) and low (scores from 0 to 4). Using Pearson's χ^2^, Fisher's exact test, ANOVA or Student's t test, where appropriate, we examined which covariates were associated with high versus low lifestyle scores.

We used log binomial regression to estimate the prevalence ratios (PR, for cross-sectional analyses), relative risks (RR, for longitudinal analyses) and 95% confidence intervals (CI) of having a high healthy lifestyle score compared with a low healthy lifestyle score for education level (participant and parental) and educational mobility. Log binomial regression is preferable to the more commonly used logistic regression because odds ratios are known to overestimate the true effect when the outcome is not rare [27]. We also examined whether participant education level modified the association between parental education and the lifestyle score by entering an interaction term between the participant and parental education into the regression model. We examined differences between categories using post-hoc Wald tests. We present models that are unadjusted and adjusted for confounding factors (Adjusted 1). To examine whether parental education was independently associated with the lifestyle score we adjusted for participant's education level (Adjusted 2).

Covariates were included in the models if they were associated with the outcome and caused at least a 10% change in the parameter estimate when included in the model. The covariates included in the final models are listed in the footnotes for each table.

We explored the impact of loss to follow-up on our results using three methods. We compared participants with non-participants using data that were collected in 1985 and also with the Australian population of a similar age, as described above. We then conducted sensitivity analyses using inverse probability weighting for variables from 1985 that differed between participants and non-participants (area-level SES, overweight and regular smoking) and we examined the differences in the magnitude of effect between the weighted and unweighted results.

We also examined whether dichotomising the healthy lifestyle score at the median had affected our results. The data were re-analysed comparing those with lifestyle scores in the highest third (scores 7 to 10) versus those with scores in the two lower thirds (scores 0 to 6). Then we analysed the data using linear regression using the healthy lifestyle score as a continuous variable.

Participants gave their informed consent and the Southern Tasmania Health and Medical Human Research Ethics Committee approved the study.

## Results

The characteristics of participants, including the distribution of education and educational mobility is shown in Table 1. The education level of participants was associated with more healthy behaviours than parental education (Table 2, Table 3, Table 4). Those in the low group, for participants or parents, were least likely to have healthy behaviours and those in the high group were most likely to have healthy behaviours. For educational mobility, the upwardly mobile group had a similar prevalence of healthy behaviours to the stable high group. A similar pattern was evident for the downwardly mobile and stable low categories.

**Table 1:** Characteristics of participants

		Males		Females	
		n	(%)	n	(%)
Mean (SD) age, years		32 ± 3		31 ± 3	
Marital status	Single	304	(33)	334	(32)
	Married/living as married	628	(67)	707	(68)
Area of residence*	Major city	744	(80)	803	(77)
	Inner regional	125	(13)	154	(15)
	Outer regional/remote	63	(7)	82	(8)
History of CVD or diabetes*		79	(9)	40	(4)
Family history of CVD or diabetes		92	(10)	107	(10)
Participant's education	High	380	(41)	511	(49)
	Intermediate	331	(36)	259	(25)
	Low	221	(24)	271	(26)
Highest of parent's education	High	285	(31)	302	(29)
	Intermediate	302	(32)	364	(35)
	Low	345	(37)	375	(36)
Educational mobility	Stable high	175	(19)	213	(21)
	Stable intermediate	134	(14)	124	(12)
	Stable low	121	(13)	141	(14)
	Downward	171	(18)	180	(17)
	Upward	331	(36)	383	(37)

* missing data: n = 2 for area of residence, n = 89 for history of CVD or diabetes

CVD: cardiovascular disease

**Table 2:** Prevalence (%) of healthy lifestyle score items by education categories in males

	Non- Smoker	BMI <25 kg/m^2^	LTPA >3 hrs/week	Alcohol <20 g/day	Low salt use	Uses skim milk	Fish ≥ 2 times/week	Meat <5 times/week	Fruit/veg. ≥ 7 times/week	Uses low fat spread
**Parent's education**										
High	58	48	39	84	25	48	50	19	8	79
Intermediate	61	41	36	85	29	45	50	14	8	70
Low	54	30	36	86	24	44	49	16	6	76
*P-value*	*0.17*	*<0.01*	*0.63*	*0.84*	*0.211*	*0.63*	*0.96*	*0.28*	*0.52*	*0.05*
**Participant's education**										
High	68	46	46	85	30	50	50	21	10	79
Intermediate	54	31	32	84	24	44	50	15	6	70
Low	43	39	29	86	22	41	48	10	6	76
*P-value*	*<0.01*	*<0.01*	*<0.01*	*0.92*	*0.08*	*0.10*	*0.93*	*<0.01*	*0.10*	*0.02*
**Educational** **mobility**										
Stable high	66	50	42	84	27	50	49	23	10	81
Stable intermediate	62	31	30	82	24	40	51	14	5	67
Stable low	45	31	27	85	17	39	43	8	7	79
Downward	44	47	32	86	26	42	52	11	6	73
Upward	63	36	43	86	29	49	51	19	8	75
*P-value*	*<0.01*	*<0.01*	*0.02*	*0.86*	*0.15*	*0.11*	*0.62*	*0.01*	*0.63*	*0.06*

Values are percentages

BMI: body mass index, LTPA: moderate to vigorous intensity leisure time physical activity

The number of participants ranges from n = 1042 to n = 1283

P-values are from Person's χ^2 ^tests

**Table 3:** Prevalence (%) of healthy lifestyle score items by education categories in females

	Non- Smoker	BMI <25 kg/m^2^	LTPA >3 hrs/week	Alcohol <20 g/day	Low salt use	Uses skim milk	Fish ≥ 2 times/week	Meat <5 times/week	Fruit/veg. ≥ 7 times/week	Uses low fat spread
**Parent's education**										
High	59	71	30	91	31	59	54	36	16	75
Intermediate	57	57	26	93	34	57	43	26	11	76
Low	53	61	29	94	33	57	46	25	11	75
*P-value*	*0.22*	*<0.01*	*0.40*	*0.42*	*0.66*	*0.92*	*0.02*	*<0.01*	*0.06*	*0.98*
**Participant's education**										
High	67	70	34	92	34	62	48	33	16	77
Intermediate	48	51	23	92	31	58	47	28	10	76
Low	42	58	21	94	33	50	45	22	9	72
*P-value*	*<0.01*	*<0.01*	*<0.01*	*0.59*	*0.76*	*<0.01*	*0.67*	*<0.01*	*<0.01*	*0.28*
**Educational mobility**										
Stable high	66	73	33	91	32	61	55	39	18	77
Stable intermediate	53	44	23	93	32	57	43	28	12	74
Stable low	40	60	18	94	33	51	44	21	9	72
Downward	44	59	24	93	33	51	47	24	8	74
Upward	63	65	32	93	33	62	46	28	13	77
*P-value*	*<0.01*	*<0.01*	*<0.01*	*0.89*	*0.99*	*0.04*	*0.17*	*<0.01*	*0.03*	*0.67*

Values are percentages

BMI: body mass index, LTPA: moderate to vigorous intensity leisure time physical activity

The number of participants ranges from n = 1112 to n = 1603

P-values are from Person's χ^2 ^tests

**Table 4:** Prevalence (%) of having > 4 healthy behaviours in males and females

	>4 healthy items (%)*	
	Males	Females
**Parent's education**		
High	63	78
Intermediate	60	70
Low	52	73
*P-value*	*0.011*	*0.079*
**Participant's education**		
High	67	81
Intermediate	53	68
Low	51	66
*P-value*	*<0.001*	*<0.001*
**Educational mobility**		
Stable high	68	83
Stable intermediate	53	66
Stable low	43	70
Downward	57	65
Upward	60	77
*P-value*	*<0.001*	*<0.001*

* Scores were dichotomised at the median value of 5

Values are percentages

*P*-values are from Person's χ^2 ^tests

Males, n = 918; females, n = 1033

Males and females with an intermediate or low level of education were significantly less likely to have a high healthy lifestyle score than those with a high level of education (Table 5). Males with a low level of parental education were less likely to have a high healthy lifestyle score than those with a high level of parental education (Table 5). Further adjustment for the participant's level of education attenuated the association in males and it was no longer significant. There was no association between parental education and having a high healthy lifestyle score in females.

**Table 5:** Relative risk of having a high* healthy lifestyle score by education level

	Unadjusted			Adjusted 1			Adjusted 2		
	RR/PR	(95% CI)	P-value	RR/PR	(95% CI)	P-value	RR/PR	(95%CI)	P-value
*Males*									
**Participant's education**									
High	1.00						----		
Intermediate	0.79	(0.69, 0.89)	<0.01	0.80	(0.70, 0.90)	<0.01	----	----	----
Low	0.76	(0.65, 0.88)	<0.01	0.76	(0.66, 0.89)	<0.01	----	----	----
**Parents' education**									
High	1.00	Reference		1.00	Reference		1.00	Reference	
Intermediate	0.94	(0.83, 1.07)	0.35	0.95	(0.84, 1.08)	0.44	1.01	(0.88, 1.14)	0.93
Low	0.82	(0.71, 0.94)	<0.01	0.84	(0.73, 0.96)	0.01	0.91	(0.78, 1.04)	0.17
*Females*									
**Participant's education**									
High	1.00			1.00			----		
Intermediate	0.85	(0.77, 0.93)	<0.01	0.85	(0.77, 0.93)	<0.01	----	----	----
Low	0.81	(0.74, 0.90)	<0.01	0.84	(0.76, 0.92)	<0.01	----	----	----
**Parents' education**									
High	1.00	Reference		1.00	Reference		1.00	Reference	
Intermediate	0.90	(0.82, 0.99)	0.02	0.93	(0.85, 1.02)	0.12	0.97	(0.89, 1.07)	0.53
Low	0.94	(0.86, 1.02)	0.14	0.97	(0.88, 1.05)	0.44	1.00	(0.92, 1.09)	0.99

* high = scores 5 to 10

RR: relative risk, PR: prevalence ratio, CI: confidence interval

Participant's education adjusted 1 includes: age for males and age, marital status and area of residence for females.

Parents' education adjusted 1 includes age for males and age, marital status and area of residence for females. Adjusted 2 models include additional adjustment for participant's education level.

RR/PR were estimated using log binomial regression models

Males, n = 918; females, n = 1033

Educational mobility was significantly associated with the healthy lifestyle score in both sexes (Table 6). The interaction terms between parental and participant education were not significant for either sex (data not shown). For both sexes, those in the stable intermediate, stable low and downwardly mobile categories were least likely to have a high healthy lifestyle score after adjustment for covariates. Post-hoc Wald tests in males indicated that the stable low group was less likely to have a high healthy lifestyle score than the upwardly (p < 0.001) and downwardly (p = 0.03) mobile groups. We were concerned that the significant difference between the downwardly mobile and stable low groups might have been due to downward mobility between the higher education categories (i.e. from high to intermediate education). To examine this we created a variable with all nine combinations of mobility (see Additional file 1 for tables). This demonstrated that those who moved from intermediate to low and high to low were significantly more likely to have a high healthy lifestyle score compared to the stable low group (data not shown). In females, post-hoc Wald tests indicated that the stable intermediate (p = 0.02) and downwardly mobile (p = 0.009) groups were less likely than the upwardly mobile group to have a high healthy lifestyle score.

**Table 6:** Relative risk of having a high* healthy lifestyle score by educational mobility

	Unadjusted			Adjusted		
	RR	(95% CI)	p-value	RR	(95% CI)	p-value
Males						
Stable high	1.00	Reference		1.00	Reference	
Stable intermediate	0.78	(0.64, 0.94)	0.009	0.80	(0.66, 0.97)	0.022
Stable low	0.62	(0.49, 0.78)	<0.001	0.64	(0.50, 0.81)	<0.001
Downward	0.83	(0.70, 0.98)	0.027	0.84	(0.72, 0.99)	0.045
Upward	0.88	(0.77, 1.01)	0.070	0.91	(0.79, 1.04)	0.167
Females						
Stable high	1.00	Reference		1.00	Reference	
Stable intermediate	0.79	(0.69, 0.92)	0.002	0.81	(0.70, 0.93)	0.003
Stable low	0.84	(0.74, 0.96)	0.007	0.87	(0.77, 0.99)	0.037
Downward	0.79	(0.69, 0.89)	<0.001	0.81	(0.71, 0.91)	0.001
Upward	0.93	(0.85, 1.01)	0.080	0.95	(0.87, 1.03)	0.209

* high = scores 5 to 10

RR: relative risk, PR: prevalence ratio, CI: confidence interval

Adjusted models include age and area of residence for both sexes

RR were estimated using log binomial regression models

Males, n = 918; females, n = 1033

The sensitivity analyses showed that weighting for overweight status made only small changes to the prevalence ratios for all exposures (educational mobility and participant or parental education) in males (range = 1% to 7%) and females (range = -2% to 8%). Weighting for area-level SES increased estimates in males (range = 20% to 50%) and females (range = 1% to 28%), with the exception of the estimate for intermediate participant education in females, which decreased by 3%. Weighting by smoking status mainly increased the estimates (males = 2% to 23% and females = 3% to 33%). However, for males there was one decrease in the intermediate parental group (69%) and in females, the estimates were reduced for the low participant education group (14%) and, in the mobility analysis, the stable low (5%) and downwardly mobile groups (5%).

Sensitivity analyses conducted by re-analysing the data comparing those with scores in the top third (score 7 to 10) to the two lower thirds (scores 0 to 6) gave results showing the same patterns as when the healthy lifestyle score was dichotomised at the median, though the magnitude of effect was greater. The results using linear regression also showed the same patterns. These results are available on request from the authors.

## Discussion

The level of education achieved predicted the adoption of not just individual health risk behaviours, but multiple healthy behaviours in this cohort of young Australian adults. The effect was stronger for the participants' levels of education than that of their parents. A change in the level of education from one generation to the next was also significantly associated with a high healthy lifestyle score, with these young adults adopting the lifestyles associated with the level of education that they attained.

At the level of specific health risk behaviours, education was associated with BMI, LTPA, smoking, and some aspects of diet, which is consistent with other studies [12,28-31]. It is difficult to compare our findings regarding overall lifestyle with other studies because few have reported on multiple risk factors and have generally used measures of occupation rather than education [12,28,29,31]. Parental education was not independently associated with having a high healthy lifestyle score. Although others have reported an independent effect of childhood SES with mortality [8], the results regarding health behaviours are more mixed. Parental occupation has been reported to be independently associated with smoking [12,32-34], obesity [2,34], alcohol consumption [12,32,35,36], and having multiple unhealthy risk factors [37]. However, others have failed to find such an association [2,12,30,32], including our recent analyses concerning changes in physical activity and fitness over time [7]. Our null finding is supported by analyses where adjustment for the current level of education has removed the independent effect of parental occupation on risk behaviours [9,35]. These results may differ to those of others for several reasons. First, our cohort comprises individuals aged between 26 and 36 years in 2004 to 2006. Most studies have included people born several decades before ours from a different generation, with some exceptions [36,38,39]. Changes in socioeconomic conditions between generations may account for the null association between parental education and lifestyle in our study. Second, we were looking at a summary measure of a healthy lifestyle. Parental education was associated with some items from the healthy lifestyle score in the univariable analyses. We suggest that because the effect is only present for some aspects of lifestyle, the contribution of parental education to overall lifestyle declines throughout the life course as children's and parent's education levels differ. This does not appear to be the case for BMI, however, which was associated with parental education in our study and also in other studies [2,34]. We suggest this is because of a confluence of two factors. First, that parental education is associated with childhood BMI [40]. Second, that obesity tracks strongly between childhood and adulthood in this cohort [14], thus linking parental education at the age of 12 to adult BMI.

The change in the level of education between parents and offspring was significantly associated with having a high healthy lifestyle score. This association was not the result of an interaction between parental and offspring education levels, i.e. the association between the education level of participants and their healthy lifestyle scores was not modified by the level of their parents' education. Other authors have also reported similar results, albeit mostly in relation to mortality [6,12,13,41]. These analyses show the strong and important role of achieved education in determining lifestyle. The weaker contribution of childhood SES, as indicated by parental education level at age 12 years, would not have been apparent without undertaking the social mobility analyses. The exception to this pattern was in males, where those moving downward were significantly more likely to have a high healthy lifestyle score than those with a stable low education. This may suggest a protective effect in males of higher parental education. However, given the large number of comparisons made we cannot rule out that this was a spurious finding. Together, these results suggest that increasing participation and achievement in education could help reduce socioeconomic inequalities in health.

The greatest absolute differences between low and high participant education or, for mobility, stable high or low groups, were for smoking, meat consumption and, less consistently, BMI. This has implications for the diseases that may show socioeconomic disparities in the future in our cohort. In relation to diseases, the differences for BMI, smoking and meat consumption suggest that cardiovascular disease, diabetes and cancers may occur more often in those with lower educational attainment. Our data suggest that interventions targeted at smoking, weight and meat consumption could have an impact on socioeconomic inequalities in health, at least among the current generation of young Australian adults. The influence of such changes on socioeconomic inequalities in health would vary depending on the strength of the association between a behaviour and disease. Furthermore, it remains uncertain whether campaigns targeted at socioeconomically disadvantaged groups are more effective than population-based campaigns to change health behaviours [42].

This study has several limitations. The loss to follow-up was considerable but we found that our sample was similar to the general population for several key health behaviours. Furthermore, the inverse probability weighting analyses demonstrated that we most likely underestimated the magnitude of effect by having a cohort with higher SES and more favourable health behaviours in 1985. As noted by others [43], the applicability of education categories over time should also be considered. For instance, between our parental and participant generations, there was a 130% increase in the completion of secondary school in Australia [44]. Even within the participants' generation, the completion of secondary school rose from 53% in 1987 to 72% in 1997, when the youngest participants finished school [22]. We accept that our groupings may not capture the true extent of educational mobility in Australia, but have used these groupings to align with other studies. We used the highest level of parental education rather than paternal education because other researchers have shown that both paternal and maternal SES are important for the health of offspring [39]. Participants retrospectively recalled parental education. Such recall is moderately accurate over a much longer period than the 14 to 24 years in our study [45], particularly for parental education [46]. Education is more straightforward to describe and stable over time than occupation [47], therefore increasing its accurate recall. Although using a younger cohort has probably limited reverse causation between lifestyle and education, we cannot discount that this may have occurred. Some researchers have shown that adolescents who take up smoking have worse educational outcomes suggesting that even in younger people lifestyle may affect SES [48].

While we believe the healthy lifestyle score has strengths, it also has limitations. We acknowledge that each item in the score does not contribute equally to the burden of disease. While it would be possible to derive weights for items based on their contribution to disease, this would make the score less accessible to the general population. Further, the association between the healthy lifestyle score and cardiovascular risk factors in this cohort [11] and its prediction of mortality [10,49] demonstrate the validity of the current scoring method. We and others have dichotomised the score for analysis purposes. This is unlikely to be suitable for clinical or public health settings where a focus on improving all health behaviours, and achieving a high healthy lifestyle score, is desirable. Finally, our analyses of the healthy lifestyle score did not specifically take into account that some of the component behaviours more often co-occur than others. Such analyses were beyond the scope of this paper given that over 400 different combinations of the 10 healthy items were present in this cohort.

The study also has several strengths. Despite the loss to follow-up the sample was large and had considerable heterogeneity of exposures and outcomes. The use of the healthy lifestyle score adds strength because this is the only study of this kind to have used a composite measure of lifestyles that predicts mortality. The examination of individual health behaviours and overall lifestyle within a single cohort also makes this study novel. Few studies of socioeconomic inequalities in health have been conducted in Australia, particularly among men.

## Conclusion

A corollary of our data is that increasing participation in education may have a positive impact on health-related behaviours. It is important to determine what it is about achieving higher education that leads to a healthy lifestyle. If it is cognitive function [9,35] or intelligence [36] then we may have less ability to intervene. Findings, mostly pertaining to the role of maternal education in infant mortality, suggest various modifiable factors account for the association between education and health outcomes. These include increasing knowledge about health, but also increasing autonomy, social support, and the ability to understand and apply information [50]. Education also strongly predicts occupation and, therefore, income. This may improve access to healthier foods or leisure facilities. Education and occupation also affect peer group and the social norms to which people are exposed, which can determine behaviours [51].

Finally, the educational disparities in lifestyle observed are concerning because the healthy lifestyle score is associated with biomedical cardiovascular risk factors in this cohort [11] and predicts mortality in older individuals [10]. Although these individuals are currently young and healthy, the findings suggest that socioeconomic inequalities in morbidity and mortality will persist in Australia for some decades to come.

## Competing interests

The authors declare that they have no competing interests.

## Authors' contributions

SLG conceived and designed the study, analysed and interpreted the data and drafted the manuscript; JAC assisted with interpretation of the data and drafted and revised the manuscript; GCP assisted with interpretation of the data and drafted and revised the manuscript; TD assisted with interpretation of the data and drafted and revised the manuscript. All authors approved the final version of the manuscript.

## Pre-publication history

The pre-publication history for this paper can be accessed here:

http://www.biomedcentral.com/1471-2458/10/55/prepub

## Supplementary Material

Additional file 1 **Association between 9 educational mobility trajectories and high healthy lifestyle scores in males and females**. Two tables containing results of statistical analyses. Click here for file
