# Supplementary material for: Intergenerational educational mobility is associated with cardiovascular disease risk behaviours in a cohort of young Australian adults: The Childhood Determinants of Adult Health (CDAH) Study
Source: BMC Public Health. 2010 Feb 2;10:55. doi: 10.1186/1471-2458-10-55 (PMC2835686; doi:10.1186/1471-2458-10-55)
Supplement: Additional file 1 — Association between 9 educational mobility trajectories and high healthy lifestyle scores in males and females. Two tables containing results of statistical analyses. [file 1471-2458-10-55-S1.DOC]

Table 1 Association between nine category educational mobility and high* healthy lifestyle scores in males

|  | **n** | **RR** | **(95% CI)** | **P-value** |
| --- | --- | --- | --- | --- |
| Stable high | 171 | 1.00 | Reference |  |
| Stable intermediate | 130 | 0.79 | (0.65, 0.96) | 0.018 |
| Stable low | 120 | 0.63 | (0.50, 0.80) | <0.001 |
| Upward (Intermediate to high) | 106 | 1.02 | (0.87, 1.21) | 0.793 |
| Upward (Low to high) | 97 | 0.94† | (0.78, 1.13) | 0.482 |
| Upward (Low to intermediate) | 123 | 0.78 | (0.64, 0.95) | 0.015 |
| Downward (Intermediate to low) | 61 | 0.87† | (0.69, 1.09) | 0.229 |
| Downward (High to low) | 39 | 0.93† | (0.71, 1.21) | 0.569 |
| Downward (High to intermediate) | 71 | 0.78 | (0.61, 1.00) | 0.047 |

* high = scores 5 to 10

† p < 0.05 compared to stable low category using post-hoc Wald test

Adjusted for age and area of residence

RR: relative risk, CI: confidence interval

n = 918

Table 2 Association between nine category educational mobility and high* healthy lifestyle scores in females

|  | **n** | **RR** | **(95% CI)** | **P-value** |
| --- | --- | --- | --- | --- |
| Stable high | 210 | 1.00 | Reference |  |
| Stable intermediate | 122 | 0.80 | (0.69, 0.93) | 0.003 |
| Stable low | 141 | 0.87 | (0.76, 0.99) | 0.033 |
| Upward (Intermediate to high) | 147 | 0.98 | (0.88, 1.08) | 0.649 |
| Upward (Low to high) | 149 | 0.97 | (0.87, 1.07) | 0.554 |
| Upward (Low to intermediate) | 85 | 0.84 | (0.72, 0.99) | 0.038 |
| Downward (Intermediate to low) | 91 | 0.78 | (0.66, 0.93) | 0.006 |
| Downward (High to low) | 38 | 0.72 | (0.54, 0.95) | 0.018 |
| Downward (High to intermediate) | 50 | 0.90 | (0.75, 1.07) | 0.234 |

* high = scores 5 to 10

† p < 0.05 compared to stable low group using post-hoc Wald tests

Adjusted for age and area of residence

RR: relative risk, CI: confidence interval

n = 1033
